# CottonGVD: A Comprehensive Genomic Variation Database for Cultivated Cottons

**DOI:** 10.3389/fpls.2021.803736

**Published:** 2021-12-21

**Authors:** Zhen Peng, Hongge Li, Gaofei Sun, Panhong Dai, Xiaoli Geng, Xiao Wang, Xiaomeng Zhang, Zhengzhen Wang, Yinhua Jia, Zhaoe Pan, Baojun Chen, Xiongming Du, Shoupu He

**Affiliations:** ^1^Zhengzhou Research Base, State Key Laboratory of Cotton Biology, Zhengzhou University, Zhengzhou, China; ^2^Institute of Cotton Research, Chinese Academy of Agricultural Sciences (CAAS), Anyang, China; ^3^School of Computer Science and Information Engineering, Anyang Institute of Technology, Anyang, China

**Keywords:** *Gossypium*, genomics, genetics, variation, eGWAS, database

## Abstract

Cultivated cottons are the most important economic crop, which produce natural fiber for the textile industry. In recent years, the genetic basis of several essential traits for cultivated cottons has been gradually elucidated by decoding their genomic variations. Although an abundance of resequencing data is available in public, there is still a lack of a comprehensive tool to exhibit the results of genomic variations and genome-wide association study (GWAS). To assist cotton researchers in utilizing these data efficiently and conveniently, we constructed the cotton genomic variation database (CottonGVD; http://120.78.174.209/ or http://db.cngb.org/cottonGVD). This database contains the published genomic information of three cultivated cotton species, the corresponding population variations (SNP and InDel markers), and the visualized results of GWAS for major traits. Various built-in genomic tools help users retrieve, browse, and query the variations conveniently. The database also provides interactive maps (e.g., Manhattan map, scatter plot, heatmap, and linkage disequilibrium block) to exhibit GWAS and expression GWAS results. Cotton researchers could easily focus on phenotype-associated loci visualization, and they are interested in and screen for candidate genes. Moreover, CottonGVD will continue to update by adding more data and functions.

## Introduction

The cotton genus (*Gossypium*) contains four major cultivated species: two diploids, such as *G. herbaceum* (A_1_) and *G. arboreum* (A_2_), and two tetraploids, such as *G. hirsutum* [(AD)_1_] and *G. barbadense* [(AD)_2_]. Cotton fiber is not only the most important natural textile materials but also the ideal model for studying the mechanism of single-cell elongation, which has been widely concerned by both cotton breeders and plant biologists. Understanding the genomic basis of phenotypic variations of cotton is essential for guiding molecular breeding practice. In the last 10 years, cultivated tetraploid cotton genomes have been assembled by both Illumina short-read (Li et al., 2014; Liu et al., 2015; Yuan et al., 2015; Zhang et al., 2015) and PacBio long-read sequencing technology (Wang M. et al., 2019; Yang et al., 2019; Chen et al., 2020; Huang et al., 2020; Ma et al., 2021). Based on these reference genomes, researchers exhibited the landscape of genomic variation during domestication of cultivated tetraploid cottons (Yuan et al., 2021) and discovered the population differentiation within cultivated upland cotton (He et al., 2019, 2020; Dai et al., 2020). By integrating the large-scale multienvironmental trait surveys and high-density SNP markers, researchers have identified an abundance of trait-associated genomic regions (Fang et al., 2017a,b; Sun et al., 2017; Wang et al., 2017; Du et al., 2018; Ma et al., 2018; Nie et al., 2020; He et al., 2021; Yu et al., 2021). Integration and utilization of these data could accelerate functional gene cloning and molecular marker designation for targeting genetic improvement of cotton cultivars.

As mentioned earlier, the vast amount of cotton genome variation data sets have been generated by the next-generation sequencing (NGS) technology and stored in the public database. With the availability of large data, a major obstacle appears, that is, how to effectively integrate and share them with the data of cotton molecular breeding team to speed up the cotton breeding. It is also very difficult to identify key SNPs and polymorphic sites from large-scale NGS data sets, which requires a lot of computing resources. Therefore, the current SNP and InDel data sets are not user-friendly. Among other species, several genomic variation databases have been developed, including RiceVatMap for rice (Zhao et al., 2015), SorGSD for sorghum (Luo et al., 2016), PeachVar-DB for peach (Cirilli et al., 2018), CitGVD for citrus (Li et al., 2020), ZEAMAP for maize (Gui et al., 2020), and BnaGVD for rapeseed (Yan et al., 2021). Here, we set up a comprehensive cotton genomic variation database (CottonGVD).

For cotton, several cotton databases have been released previously. Cottongen^1^ is a comprehensively cotton database that integrated extensive data, including genomes, genetic maps, molecular markers, and phenotypes (Yu et al., 2014). ccNET^2^ provides the genome-scale co-expression networks with functional modules for *G. arboreum* and *G. hirsutum* genes (You et al., 2017). CottonFGD^3^ is a database that mainly focuses on collecting the genome information (Zhu et al., 2017), and COTTONOMICS^4^ is a comparative genomics platform and variation database for the tetraploid cotton genus. GRAND^5^ is also a comparative genomics platform for *Gossypium* spp. However, all these databases lack modules to exhibit genome-wide association study (GWAS) results that can show the phenotypic traits (various types) of multiple populations of different cotton species. In this study, we constructed CottonGVD (cotton genomic variation database^6^); the first cotton database specifically focuses on trait-associated loci visualization. This interface-friendly website could facilitate researchers in searching for the details of their interested genomic variations on the cotton genome.

## Data Collection and Processing

### Germplasm Populations and Data Sources

**FIGURE 1:**
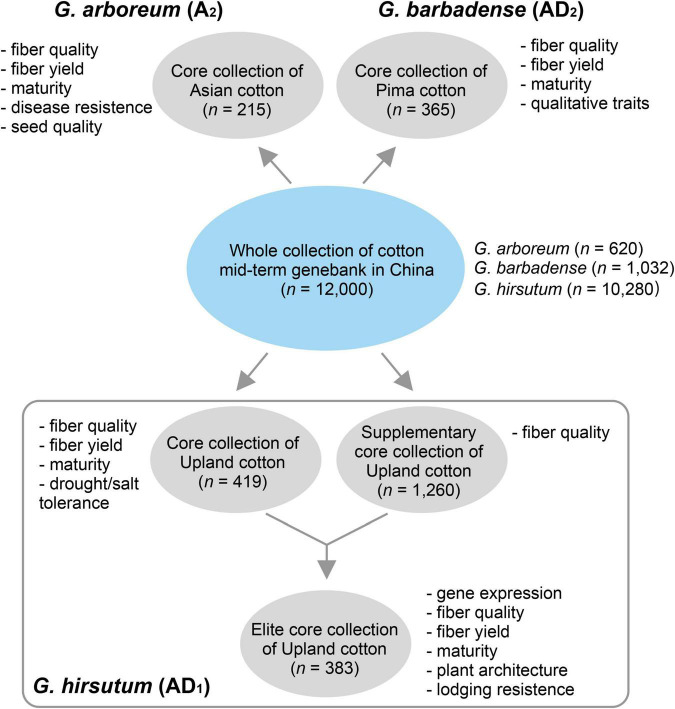
Sources of natural populations of three different cotton species selected.

In this version of database (version 1.0), we provided five germplasm populations selected from the whole germplasm collection of the National Medium-Term Gene Bank of Cotton in China (*n* = 11,000) (Figure 1). *G. arboreum* is an ancient diploid cultivated cotton that has been obsoleted in most of the cotton-producing regions worldwide. *G. barbadense* is one of the two tetraploid species with high-fiber quality but lower yield, which are grown in a limited region due to its sensitivity to photoperiod and frost. Two populations containing 215 and 365 diverse *G. arboreum* (cottonA2.215) (Du et al., 2018) and *G. barbadense* (cottonAD2.365) (unpublished data) accessions were selected as core collections, respectively. *G. hirsutum* (Upland cotton) is the most important cultivated tetraploid cotton, which produces more than 97% natural fiber in the modern world. As *G. hirsutum* contains the most abundant accessions in the genebank (*n* = 10,280), we totally selected three populations (i.e., cottonAD1.419, cottonAD1.1245, and cottonAD1.383) to represent these species from published projects (e.g., PRJNA399050, PRJNA349094, and PRJNA605345) (Ma et al., 2018; He et al., 2021; Figure 1).

### Data Sources and Processing

**FIGURE 2:**
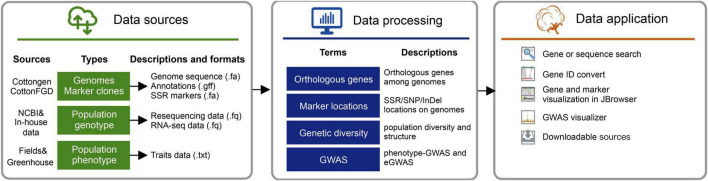
Data sources and pipelines to construct cotton genomic variation database (CottonGVD).

The raw bioinformatic sources of the initial version (version 1.0) included high-quality (PacBio-read assembles) genomes for three major cultivated cotton species^7^, including *G. arboreum* (A2_CRI), *G. hirsutum* (Gh_CRI v1), and *G. barbadense* (Gb_HAU.2). Variants (SNPs and InDels) and GWAS results obtained from the earlier five resequencing projects were launched in this database version. We also collected the population RNA-seq data (the ovule of 5-day post-anthesis) from cottonAD1.383 (PRJNA776409). And the available RNA-Seq data published along with reference genomes (PRJNA494275, PRJNA507565, and PRJNA490626) were also collected (Renny-Byfield et al., 2014; Cheng et al., 2019; Hu et al., 2019; Wang K. et al., 2019). Processed raw data were applied for variation calling and GWAS visualization *via* the in-house pipeline (Figure 2).

### Phenotypic Data Collection

All phenotypic information was collected from published references (our lab and collaborators) (Du et al., 2018; Ma et al., 2018; He et al., 2021) and unpublished studies. The ecological environment of data collection has been recorded in published literature. For cottonAD1.383 and cottonAD2.365 population, all accessions were planted in three locations, including Akesu (41.15°N, 80.29°E, Xinjiang Uygur Autonomous Region, China), Sanya (18.14°N, 109.31°E, Hainan China), and Anyang (36.08°N, 114.48°E, Henan China), with three replicates in each location. As for the location of Xinjiang, some ecological environment settings are also divided into two regions, Shihezi (44.40°N, 86.16°E), on behalf of the northern Xinjiang region. Another location in Xinjiang, Aral (40.61°N, 81.33°E), was selected to represent southern Xinjiang region. All the yield-, fiber- quality-, and maturity-related traits were investigated in two locations for 3 years.

## Database Implementation

All data in CottonGVD is stored and managed in PostgreSQL (version 12.0). The web interface is implemented with HTML5 and JavaScript (version 7.0.12), and JavaScript is also used for data visualization. The service of CottonGVD is deployed on the Apache Web server running ubuntu server 20.04. Data analysis mainly uses Python scripts.

## Database Content and Features

### Overview Structures of CottonGVD Database

CottonGVD is a user-friendly variation database of cotton (*Gossypium* spp.). The web interface of the database is designed to comprise the following seven components: Home, Species, Search, Toolbox, Help, Login, and Register. Among them, there are many shortcut tools in the “Toolbox” and “Help” drop-down menus to facilitate various needs of users. The multiomics data in CottonGVD are divided into four categories, involving the main content modules of genome, variation, genetics, and population diversity. Each functional module in CottonGVD has its own page, and functions are linked through the gene ID relationship.

### Data Mining and Discovery

**FIGURE 3:**
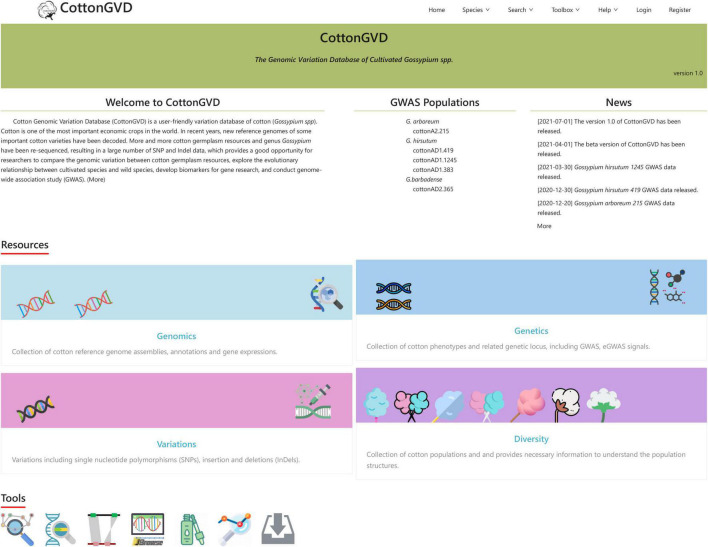
A screenshot of the CottonGVD home page.

Current CottonGVD includes four modules: (1) genomics, (2) variations, (3) genetics, and (4) diversity (Figure 3).

**FIGURE 4:**
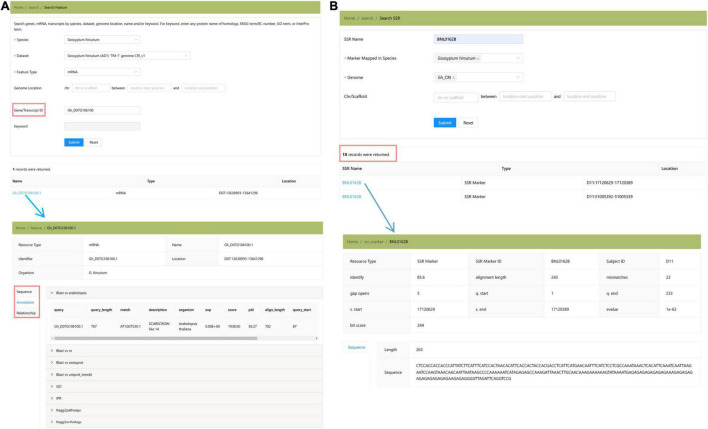
The “Genomics” module providing the summary of *Gossypium* genomes and the detailed annotation information of three species. **(A)** Search features. **(B)** Search SSR marker.

The “Genomics” module collected the genome assemblies and annotations of three cultivated cotton species (Figure 4). Users can locate the genomic position of any targeted DNA, mRNA, and protein sequence by using the “Search Feature” toolbox (Figure 4A). In addition, we provide the locations of SSR markers in Gh_CRI v1 genome in the “Search SSR Markers” toolbox, which is convenient for researchers to locate SSR markers in the newly assembled genome (Figure 4B).

**FIGURE 5:**
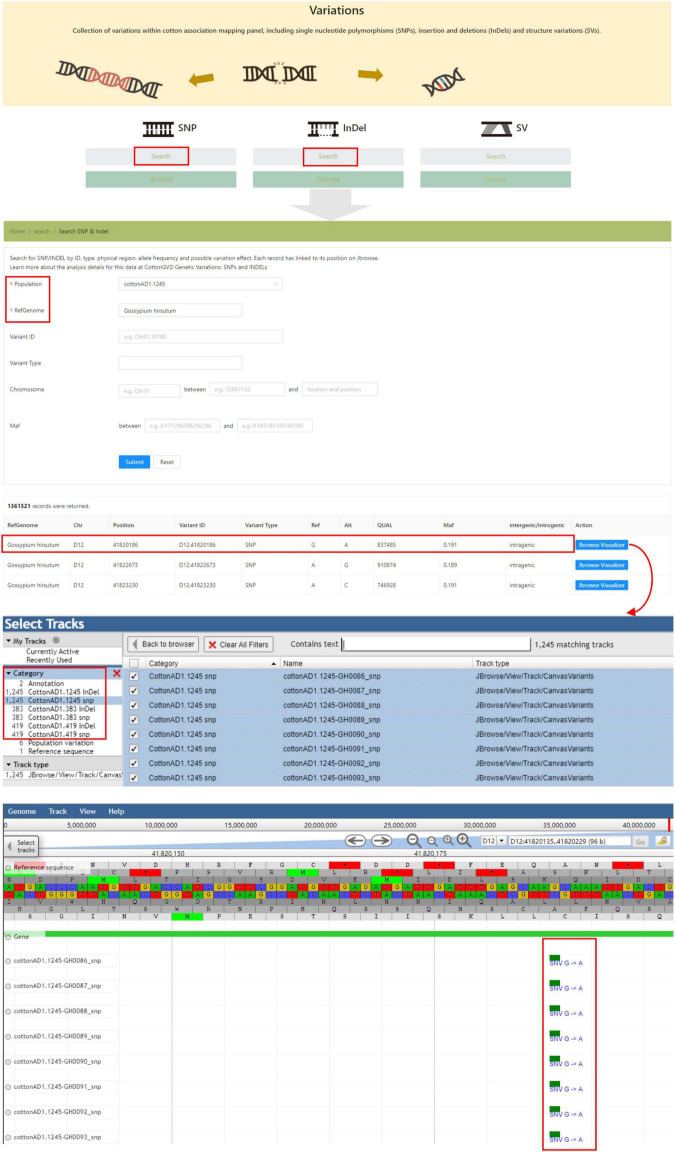
The “Variations” module providing the SNP and InDel data of five natural populations of different cotton species and genome browser tool.

The “Variations” module shows all identified polymorphic SNPs and InDels from resequencing projects. Users could search variations in the corresponding population by data type (InDel/SNP), genomic location, and allele frequency. In this interface, users could also obtain the genotypes of any selected accessions by adjusting tracks in JBrowse (Figure 5).

**FIGURE 6:**
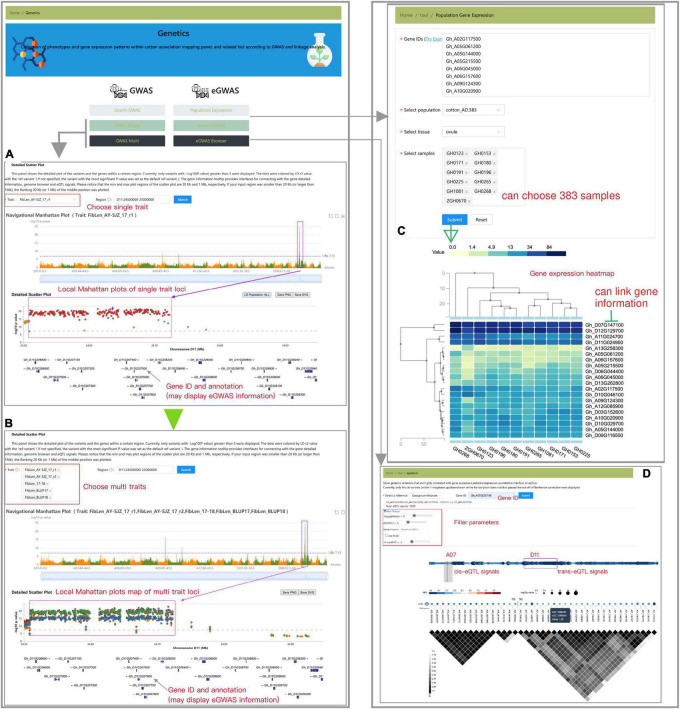
Schematic diagram of the “Genetics” module, including “GWAS-Single” **(A)**, “GWAS-Multi” **(B)**, “Population Gene Expression” **(C)**, and “eGWAS Browser” tools **(D)**.

The “Genetics” module presents the detailed GWAS result information of five cotton germplasm populations (Figure 6). By using the “search GWAS” toolbox, users could find trait-associated loci visualization by trait name, variation ID, chromosome region, or significant *p*-value. Users could access an interactive genome-wide Manhattan plot when they selected the “GWAS Single” toolbox (Figure 6A). By navigating a specific genomic region (usually the region of strongest signal), users could obtain the local Manhattan plot and select any SNP as a reference to calculate nearby linkage disequilibrium (LD) status. The “GWAS Multi” tool was designed to compare GWAS signals between two or more traits, with colors representing different traits (Figure 6B). These two tools provide a lightweight browser for genome indicating gene models in the current region. The genes included in the significance region are also interactive and linked to other relevant information, such as gene annotation and expression GWAS (eGWAS) results (if any). Each element of the graph is interactive and links to other relevant information. eGWAS mapping is an effective method to detect gene expression variation. Based on *G. hirsutum* annotations, we collected eGWAS signals with gene expression patterns [-log_10_ (*p*-value) > 7] of 5 DPA ovules of cottonAD1.383 population. Expression patterns of genes can be visualized through heatmaps *via* querying the “Population Gene Expression” button (Figure 6C). We also provide a tabular tool to search and screen eGWAS signals by gene ID, gene location, distance from transcription start site, effect size, and significance value. The visualization tool could also exhibit the significant variations (detected by eGWAS) that affect the selected genes and interactively display the significance value, effect size, and pairwise LD information (Figure 6D).

The “Diversity” module provides an interactive interface to exhibit the genetic diversity [principal component analysis (PCA) scatterplot] and population structure (stacked bars) of 3,248 sequenced tetraploid cottons which covered nearly one-third of the whole Chinese cotton gene bank (He et al., 2021). It provides the whole landscape of genetic divergence of tetraploid cotton germplasm and could be helpful for cotton evolutionary study and parent selection for breeding designation. This project provides the interactive information of 3,000 population and evaluates the population structure by PCA. We have also added a table that lists the information of origin or species characteristics for each accession of CottonGVD.

**FIGURE 7:**
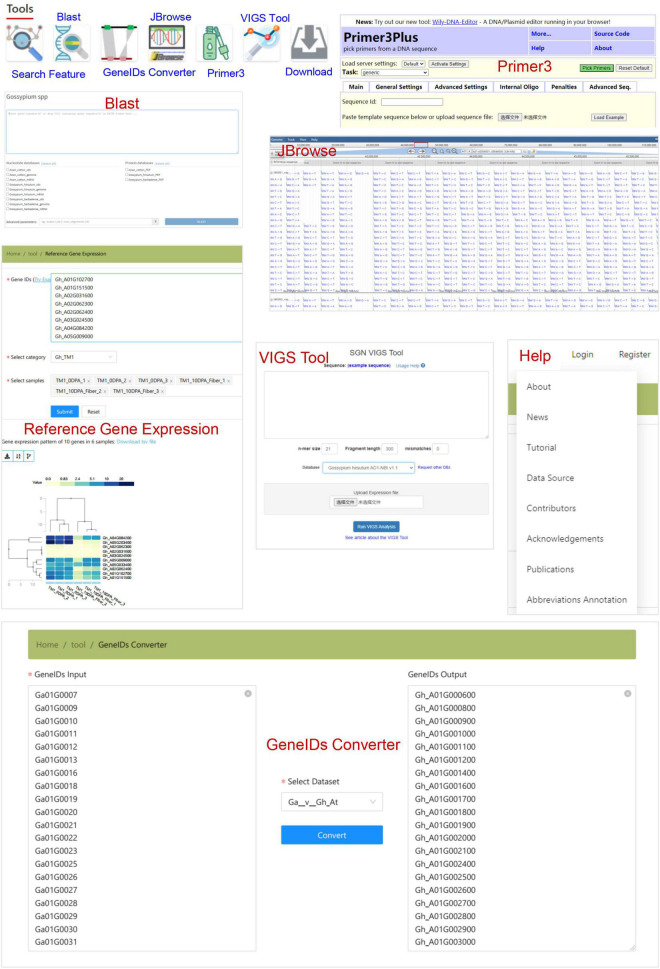
The bioinformatics tools for gene search, sequence blast, homologous gene transformation, variation data browsing, primer design, VIGS primer tool, and some genomic and phenotypic data.

In addition, CottonGVD provides some popular bioinformatics tools for search, comparison, design, and download, which are in the navigation bar under the home page (Figure 7). The “Feature search” tool allows users to enter gene information in specific format codes or keywords. The “blast” tool performs the homology search of data sets of different cotton species. The “GeneIDs Converter” tool can be used to detect gene homology among cotton species. The “JBrowse” tool provides a fast and interactive genome browser for navigating large-scale resequencing data within the genome framework. “Primer3” is a primer design tool. The “VIGS” tool sets different parameters for a given gene sequence and selects the reference genome to display the best_target_region. The “Download” section allows users to freely access all the data collected in bulk by CottonGVD. In addition, the “Reference Gene Expression” results of different tissues of SXY1, TM-1, and Hai7124 from the public database (PRJNA494275, PRJNA507565, and PRJNA490626) were provided. Finally, we provided a “Help” section to let registered users know the update status of the database, the user manual, the data sources used, and the meaning of abbreviations of phenotypic indicators.

### CottonGVD Case Study

To help researchers carry out cotton molecular breeding and effective genome-wide selection, we constructed a CottonGVD, which contains the resequencing data (SNP and InDel) of five natural populations of three different cotton species and a large number of environmental phenotype data, and visualized the results of GWAS of their phenotypes. Here, we provide an example to demonstrate the use of GWAS visualization tool. GWAS results can be obtained directly by selecting cottonAD1.1245 population and multiple environmental phenotypic data of fiber length traits in Upland cotton (He et al., 2021). Finally, Manhattan map and scatter plot with significant genetic difference loci are identified and saved. On the one hand, the list of the genes of this interval and the transcriptome data of cottonAD1.383 population and TM-1 can be obtained. On the other hand, a single gene can be selected to obtain annotation information and further explore important gene expression regulation mechanisms based on the eGWAS results.

The steps of this example are as follows.

1. After logging into CottonGVD, click the “Toolbox” drop-down menu on the top navigation bar of the home page and select “GWAS Visualizer.” Alternatively, click the middle area tab “Genetics” to enter the GWAS and eGWAS interfaces, and click any button of “GWAS single” or “GWAS multi” to enter the “GWAS Visualizer” interface (Supplementary Figure 1).
2. GWAS results can be obtained directly by choosing the “CottonAD1.1245” population and selecting GWAS Visualizer-type “GWAS Multi-Trait” (Supplementary Figure 2A). Then, once the five fiber length traits (multiyear and multipoint data) were selected, GWAS results appeared immediately in the form of Manhattan chart and scatter plot (Supplementary Figure 2B). In the Manhattan diagram of this example, it can be clearly seen that four loci are significantly superior ectopic sites [-log_10_ (*p*-value) > 7.13], namely, FL2, FL3, FL4, and FL5. Click the main peak of each locus with the mouse, and the scatter plot of the significant heterotopic loci region can be displayed below. In this way, we can obtain the chromosome position and boundary information of the significant heterotopic loci region and display the information of genes of the loci region. FL2, FL3, FL4, and FL5 were detected on chromosomes D11 (24.51–24.78 Mb), A09 (61.84–62.12 Mb), A10 (Gh_A10G233100), and A07 (88.39–88.56 Mb), respectively (Supplementary Figure 2B).
3. If we want to get the list of the genes in the FL2 genetic locus interval (Supplementary Figure 3A), we can enter the search page in the “Search” menu “Search feature” in the navigation bar of the home page, then select the species name, genome version, and type (mRNA), and finally enter the chromosome number and the position information of the start (24,510,000) and end (24,800,000) of the locus interval, and the results of the list of the genes will be returned (19 records) (Supplementary Figure 3B).
4. To understand the expression of genes in this interval in the population transcriptome (this is only suitable for Upland cotton population), we can enter the “Population Gene Expression” page in the “Toolbox” menu of the navigation bar on the home page, paste the list of 19 genes in the “Gene ID” box, select the population (cottonAD1.383) and tissue (ovule), and finally select the sample ID which we are interested in the samples box (e.g., here we can select 16 samples with good fiber quality and 16 samples with low fiber quality) (Supplementary Figure 4A). After submitting the information, the expression heatmap [fragments per kilobase of transcript per million (FPKM) value] of these 19 genes in 32 samples will be returned. At this time, we can choose to download the original table (.tsv) of expression heatmap, or cluster the samples (or not), and then obtain different expression heatmaps. The heatmap is interactive. We can click each box to display the sample name, gene ID, and FPKM value. We can also click the rightmost gene ID to connect to the annotation information and gene sequence of the gene (Supplementary Figure 4B).
5. To understand the expression patterns of 19 genes in different tissues of Upland cotton (this is applicable to Upland cotton, Asian cotton, and Sea Island Cotton populations), we can enter the “Reference gene expression” page in the “Toolbox” menu of the home navigation bar, paste the list of 19 genes into the “gene ID” box, and select the category (Gh_TM1), Finally, we can select the tissue information we are interested in the sample box (e.g., we can select 15 tissue samples (three repeats) of ovules, fibers, roots, stems, leaves, etc.) (Supplementary Figure 5A). After submitting the information, the expression heatmap (FPKM value) of these 19 genes in 45 tissue samples will be returned. The same operation is described earlier (Supplementary Figure 5B).
6. In another way, if we click the gene ID under the scatter plot displayed by the significant locus, the annotation information of the gene will appear. If the gene appears in the eGWAS result of Upland cotton, there will also be a hyperlink to the eGWAS result page of the gene. For example, according to the earlier steps, we found that the possible candidate gene in the FL3 locus interval related to fiber length is Gh_A09G105000 (to know more about this gene, refer to Supplementary Figure 6A). The first way is to directly click the gene ID in the heatmap in the previous step to hyperlink to the annotation page of this gene. The other way is to display the gene names of different genomic positions under the scatter plot, and then click Gh_A09G105000 to display the ID and transcript ID of the gene, the annotation information of *Arabidopsis* database, and eGWAS information (if there are eGWAS results of this gene in this database). After clicking the gene, it will be linked to the sequence information and related to mRNA information of the gene, and then click mRNA ID to obtain the annotation information of the gene in the seven types of database (Supplementary Figure 6B). Clicking the eGWAS Visualizer gene ID will link to the eGWAS result information of 5 ovule gene expressions in cottonAD1.383 population. The eGWAS signal is displayed interactively by adjusting the significance value [-log_10_ (*p*-value)], effect size LD cutoff R^2^, and paired LD information (Supplementary Figure 6C).

## Conclusion and Future Research

By integrating the resequencing data of many cotton species and GWAS data of five representative populations, the high-density variation data displayed by CottonGVD provide a rich information for examining the genomic variation, gene annotation, and visualization of SNP and GWAS results. Users can locate causal genes in the genomic region of GWAS signals by integrating transcriptome and eGWAS results, which could further guide the targeted gene editing. This new database will promote molecular breeding by integrating high-density genomic variations in the development of molecular markers and selections for genetic improvement of yield and fiber quality with the new designing molecular approaches based on the new tool modules. In addition to the cooperation between the different scientific teams, we will also cooperate with domestic and international laboratories to resequencing more cotton germplasm resources and GWAS in future research, and will provide more resources and tool modules for this database.

## Data Availability Statement

The original contributions presented in the study are publicly available. This data can be found here: https://www.ncbi.nlm.nih.gov/bioproject/PRJNA776409.

## Author Contributions

XD and SH designed the research. XW, XZ, ZW, YJ, XG, ZhaP, and BC organized the phenotypic data. ZheP, HL, GS, and PD organized the genotype data. ZheP, HL, and SH constructed the database and wrote this manuscript. All authors contributed to the article and approved the submitted version.

## Conflict of Interest

The authors declare that the research was conducted in the absence of any commercial or financial relationships that could be construed as a potential conflict of interest.

## Publisher’s Note

All claims expressed in this article are solely those of the authors and do not necessarily represent those of their affiliated organizations, or those of the publisher, the editors and the reviewers. Any product that may be evaluated in this article, or claim that may be made by its manufacturer, is not guaranteed or endorsed by the publisher.

## Footnotes

1
http://www.cottongen.org/

2
http://structuralbiology.cau.edu.cn/gossypium/

3
https://cottonfgd.org/

4
http://cotton.zju.edu.cn/

5
http://grand.cricaas.com.cn/home

6
http://120.78.174.209

7
https://www.cottongen.org/data/download

## Supplementary Material

Supplementary Figure 1 Two ways to enter the genome-wide association study (GWAS) Visualizer tool interface are shown.

Supplementary Figure 2 GWAS analysis steps of multienvironmental traits of cottonAD1.1245 population.

Supplementary Figure 3 Confirmation of the interval of fiber length related to favorable allele FL2 and acquisition of list of the genes.

Supplementary Figure 4 Analysis of the expression pattern of candidate genes in the FL2 heterotopic region related to fiber length in cottonAD1.383 population transcriptome data.

Supplementary Figure 5 Analysis of the expression pattern of candidate genes in the FL2 heterotopic region related to fiber length in different tissues of reference genome TM-1 transcriptome data.

Supplementary Figure 6 Search for genes/proteins sequence information, gene function annotation and eGWAS information of the FL3 favorable allele associated with fiber length.

## References

[B1] Chen Z. J. Sreedasyam A. Ando A. Song Q. De Santiago L. M. Hulse-Kemp A. M. (2020). Genomic diversifications of five Gossypium allopolyploid species and their impact on cotton improvement. *Nat. Genet.* 52 525–533. 10.1038/s41588-020-0614-5 32313247 PMC7203012

[B2] Cheng H. Sun G. He S. Gong W. Peng Z. Du X. (2019). Comparative effect of allopolyploidy on transposable element composition and gene expression between *Gossypium hirsutum* and its two diploid progenitors. *J. Integr. Plant Biol.* 61 45–59. 10.1111/jipb.12763 30565413

[B3] Cirilli M. Flati T. Gioiosa S. Tagliaferri I. Ciacciulli A. Bottoni P. (2018). PeachVar-DB: a curated collection of genetic variations for the interactive analysis of peach genome data. *Plant Cell Physiol.* 59:e2. 10.1093/pcp/pcx183 29216377

[B4] Dai P. Sun G. Jia Y. Pan Z. Tian Y. Du X. (2020). Extensive haplotypes are associated with population differentiation and environmental adaptability in Upland cotton (*Gossypium hirsutum*). *Theor. Appl. Genet.* 133 3273–3285. 10.1007/s00122-020-03668-z 32844253

[B5] Du X. Huang G. He S. Yang Z. Sun G. Liu M. (2018). Resequencing of 243 diploid cotton accessions based on an updated a genome identifies the genetic basis of key agronomic traits. *Nat. Genet.* 50 796–802. 10.1038/s41588-018-0116-x 29736014

[B6] Fang L. Gong H. Hu Y. Liu C. Zhou B. Du X. (2017a). Genomic insights into divergence and dual domestication of cultivated allotetraploid cottons. *Genome Biol.* 18 1–13. 10.1186/s13059-017-1167-5 28219438 PMC5317056

[B7] Fang L. Wang Q. Hu Y. Jia Y. Chen J. Zhou B. (2017b). Genomic analyses in cotton identify signatures of selection and loci associated with fiber quality and yield traits. *Nat. Genet.* 49 1089–1098. 10.1038/ng.3887 28581501

[B8] Gui S. Yang L. Li J. Luo J. Yan J. (2020). ZEAMAP, a comprehensive database adapted to the maize multi-omics era. *iScience* 23:101241. 10.1016/j.isci.2020.101241 32629608 PMC7306594

[B9] He S. Sun G. Geng X. Gong W. Dai P. Wang L. (2021). The genomic basis of geographic differentiation and fiber improvement in cultivated cotton. *Nat. Genet.* 53 916–924. 10.1038/s41588-021-00844-9 33859417

[B10] He S. Sun G. Huang L. Yang D. Dai P. Wei S. (2019). Genomic divergence in cotton germplasm related to maturity and heterosis. *J. Integr. Plant Biol.* 61 929–942. 10.1111/jipb.12723 30253066

[B11] He S. Wang P. Zhang Y. M. Dai P. Nazir M. F. Wang L. (2020). Introgression leads to genomic divergence and responsible for important traits in upland cotton. *Front. Plant Sci.* 11:929. 10.3389/fpls.2020.00929 32774337 PMC7381389

[B12] Hu Y. Chen J. Fang L. Zhang Z. Ma W. Lian J. (2019). Gossypium barbadense and *Gossypium hirsutum* genomes provide insights into the origin and evolution of allotetraploid cotton. *Nat. Genet.* 51 739–748. 10.1038/s41588-019-0371-5 30886425

[B13] Huang G. Wu Z. Percy R. G. Bai M. Li Y. Zhu Y. (2020). Genome sequence of Gossypium herbaceum and genome updates of *Gossypium arboreum* and *Gossypium hirsutum* provide insights into cotton A-genome evolution. *Nat. Genet.* 52 516–524. 10.1038/s41588-020-0607-4 32284579 PMC7203013

[B14] Li F. Fan G. Wang K. Sun F. Yuan Y. Zou C. (2014). Genome sequence of the cultivated cotton *Gossypium arboreum*. *Nat. Genet.* 46 567–572. 10.1038/ng.2987 24836287

[B15] Li Q. Qi J. Qin X. Dou W. He Y. (2020). CitGVD: a comprehensive database of citrus genomic variations. *Hortic. Res.* 7:12. 10.1038/s41438-019-0234-3 32025315 PMC6994598

[B16] Liu X. Zhao B. Zheng H. J. Hu Y. Lu G. Zhang L. (2015). Gossypium barbadense genome sequence provides insight into the evolution of extra-long staple fiber and specialized metabolites. *Sci. Rep.* 5:14139. 10.1038/srep14139 26420475 PMC4588572

[B17] Luo H. Zhao W. Wang Y. Xia Y. Wu X. Zhang L. (2016). SorGSD: a sorghum genome SNP database. *Biotechnol. Biofuels* 9:6. 10.1186/s13068-015-0415-8 26744602 PMC4704391

[B18] Ma Z. He S. Wang X. Sun J. Zhang Y. Sun G. (2018). Resequencing a core collection of upland cotton identifies genomic variation and loci influencing fiber quality and yield. *Nat. Genet.* 50 803–813. 10.1038/s41588-018-0119-7 29736016

[B19] Ma Z. Zhang Y. Wu L. Zhang G. Sun Z. Liu Z. (2021). High-quality genome assembly and resequencing of modern cotton cultivars provide resources for crop improvement. *Nat. Genet.* 53 1385–1391. 10.1038/s41588-021-00910-2 34373642 PMC8423627

[B20] Nie X. Wen T. Shao P. Tang B. Nuriman-guli A. Lin Z. (2020). High-density genetic variation maps reveal the correlation between asymmetric interspecific introgressions and improvement of agronomic traits in Upland and Pima cotton varieties developed in Xinjiang, China. *Plant J.* 103 677–689. 10.1111/tpj.14760 32246786 PMC7496985

[B21] Renny-Byfield S. Gallagher J. P. Grover C. E. Szadkowski E. Page J. T. Wendel J. F. (2014). Ancient gene duplicates in Gossypium (cotton) exhibit near-complete expression divergence. *Genome Biol. Evol.* 6 559–571. 10.1093/gbe/evu037 24558256 PMC3971588

[B22] Sun Z. Wang X. Liu Z. Gu Q. Zhang Y. Wu L. (2017). Genome-wide association study discovered genetic variation and candidate genes of fibre quality traits in *Gossypium hirsutum* L. *Plant Biotechnol. J.* 15 982–996. 10.1111/pbi.12693 28064470 PMC5506648

[B23] Wang K. Wang D. Zheng X. Qin A. Zhou J. Zhou Y. (2019). Multi-strategic RNA-seq analysis reveals a high-resolution transcriptional landscape in cotton. *Nat. Commun.* 10:4714. 10.1038/s41467-019-12575-x 31624240 PMC6797763

[B24] Wang M. Tu L. Lin M. Lin Z. Wang P. Zhang L. (2017). Asymmetric subgenome selection and cis-regulatory divergence during cotton domestication. *Nat. Genet.* 49 579–587. 10.1038/ng.3807 28263319

[B25] Wang M. Tu L. Yuan D. Zhu D. Shen C. Zhao G. (2019). Reference genome sequences of two cultivated allotetraploid cottons, *Gossypium hirsutum* and *Gossypium barbadense*. *Nat. Genet.* 51 224–229. 10.1038/s41588-018-0282-x 30510239

[B26] Yan T. Yao Y. Wu D. Jiang L. (2021). *BnaGVD: a Genomic Variation Database of Rapeseed (Brassica napus).* Oxford: Oxford University Press. 10.1093/pcp/pcaa169 33399824

[B27] Yang Z. Ge X. Yang Z. Qin W. Sun G. Wang Y. (2019). Extensive intraspecific gene order and gene structural variations in upland cotton cultivars. *Nat. Commun.* 10:2989. 10.1038/s41467-019-10820-x 31278252 PMC6611876

[B28] You Q. Xu W. Zhang K. Zhang L. Yi X. Provart N. J. (2017). ccNET: database of co-expression networks with functional modules for diploid and polyploid Gossypium. *Nucleic Acids Res.* 45 D1090–D1099. 28053168 10.1093/nar/gkw910 PMC5210623

[B29] Yu J. Hui Y. Chen J. Yu H. Gao X. Zhao T. (2021). Whole-genome resequencing of 240 *Gossypium barbadense* accessions reveals genetic variation and genes associated with fiber strength and lint percentage. *Theor. Appl. Genet.* 134 3249–3261. 10.1007/s00122-021-03889-w 34240238

[B30] Yu J. Jung S. Cheng C. H. Ficklin S. P. Lee T. Main D. (2014). CottonGen: a genomics, genetics and breeding database for cotton research. *Nucleic Acids Res.* 42 D1229–D1236. 10.1093/nar/gkt1064 24203703 PMC3964939

[B31] Yuan D. Grover C. E. Hu G. Pan M. Miller E. R. Wendel J. F. (2021). Parallel and intertwining threads of domestication in allopolyploid cotton. *Adv. Sci.* 8:2003634. 10.1002/advs.202003634 34026441 PMC8132148

[B32] Yuan D. Tang Z. Wang M. Gao W. Tu L. Zhu L. (2015). The genome sequence of Sea-Island cotton (*Gossypium barbadense*) provides insights into the allopolyploidization and development of superior spinnable fibres. *Sci. Rep.* 5:1766. 10.1038/srep17662 26634818 PMC4669482

[B33] Zhang T. Hu Y. Jiang W. Fang L. Guan X. Stelly D. M. (2015). Sequencing of allotetraploid cotton (*Gossypium hirsutum* L. acc. TM-1) provides a resource for fiber improvement. *Nat. Biotechnol.* 33 531–537. 10.1038/nbt.3207 25893781

[B34] Zhao H. Yao W. Ouyang Y. Yang W. Wang G. Xie W. (2015). RiceVarMap: a comprehensive database of rice genomic variations. *Nucleic Acids Res.* 43 1018–1022. 10.1093/nar/gku894 25274737 PMC4384008

[B35] Zhu T. Liang C. Meng Z. Sun G. Meng Z. Zhang R. (2017). CottonFGD: an integrated functional genomics database for cotton. *BMC Plant Biol.* 17:101. 10.1186/s12870-017-1039-x 28595571 PMC5465443

