# Supplementary material for: CottonGVD: A Comprehensive Genomic Variation Database for Cultivated Cottons
Source: Front Plant Sci. 2021 Dec 21;12:803736. doi: 10.3389/fpls.2021.803736 (PMC8724205; doi:10.3389/fpls.2021.803736)

Figure S1

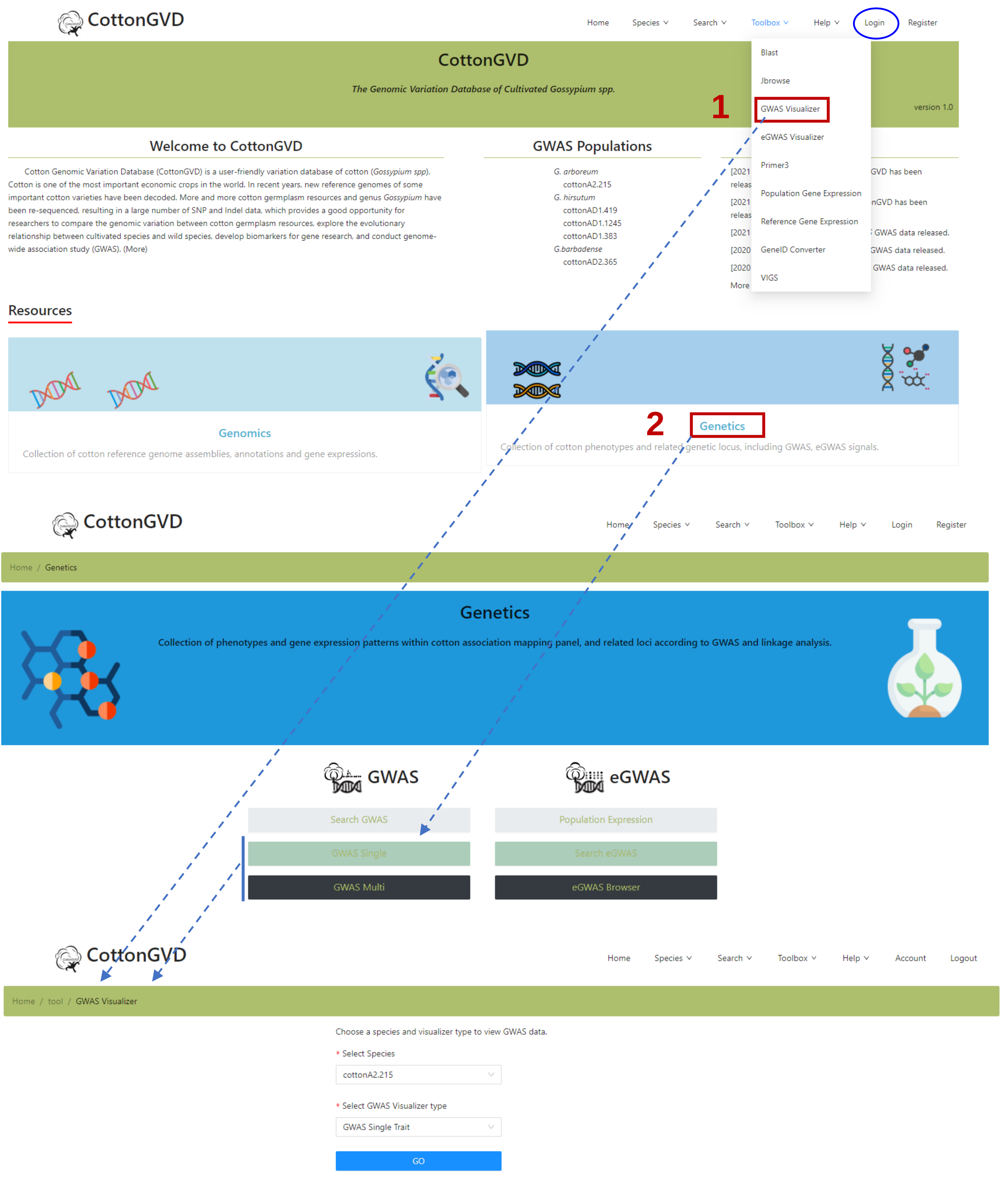

CottonGVD
Home
Species
Search
Toolbox
Help
Login
Register
CottonGVD
The Genomic Variation Database of Cultivated Gossypium spp.
version 1.0
Blast
Jbrowse
GWAS Visualizer
eGWAS Visualizer
Primer3
Population Gene Expression
Reference Gene Expression
GeneID Converter
VIGS
1
Welcome to CottonGVD
Cotton Genomic Variation Database (CottonGVD) is a user-friendly variation database of cotton (Gossypium spp). Cotton is one of the most important economic crops in the world. In recent years, new reference genomes of some important cotton varieties have been decoded. More and more cotton germplasm resources and genus Gossypium have been re-sequenced, resulting in a large number of SNP and Indel data, which provides a good opportunity for researchers to compare the genomic variation between cotton germplasm resources, explore the evolutionary relationship between cultivated species and wild species, develop biomarkers for gene research, and conduct genome-wide association study (GWAS). (More)
GWAS Populations
G. arboreum
cottonA2.215
G. hirsutum
cottonAD1.419
cottonAD1.1245
cottonAD1.383
G.barbadense
cottonAD2.365
Resources
Genomics
Collection of cotton reference genome assemblies, annotations and gene expressions.
2
Genetics
Collection of cotton phenotypes and related genetic locus, including GWAS, eGWAS signals.
CottonGVD
Home
Species
Search
Toolbox
Help
Login
Register
Home / Genetics
Genetics
Collection of phenotypes and gene expression patterns within cotton association mapping panel, and related loci according to GWAS and linkage analysis.
GWAS
eGWAS
Search GWAS
Population Expression
GWAS Single
Search eGWAS
GWAS Multi
eGWAS Browser
CottonGVD
Home
Species
Search
Toolbox
Help
Account
Logout
Home / tool / GWAS Visualizer
Choose a species and visualizer type to view GWAS data.
* Select Species
cottonA2.215
* Select GWAS Visualizer type
GWAS Single Trait
GO

Figure S2

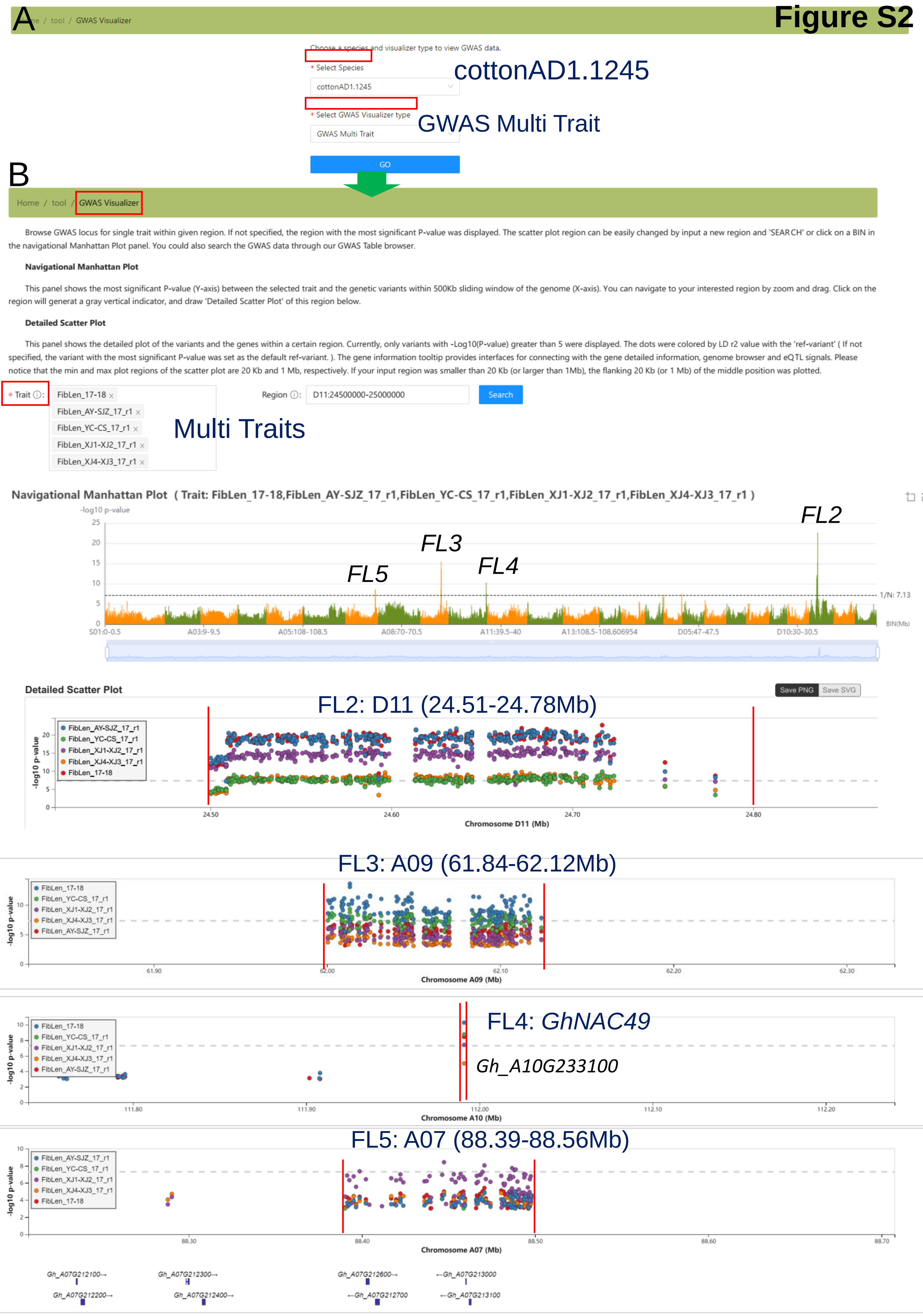

A
tool / GWAS Visualizer
Choose a species and visualizer type to view GWAS data.
* Select Species
cottonAD1.1245
cottonAD1.1245
* Select GWAS Visualizer type
GWAS Multi Trait
GWAS Multi Trait
GO
B
Home / tool / GWAS Visualizer
Browse GWAS locus for single trait within given region. If not specified, the region with the most significant P-value was displayed. The scatter plot region can be easily changed by input a new region and 'SEARCH' or click on a BIN in the navigational Manhattan Plot panel. You could also search the GWAS data through our GWAS Table browser.
Navigational Manhattan Plot
This panel shows the most significant P-value (Y-axis) between the selected trait and the genetic variants within 500Kb sliding window of the genome (X-axis). You can navigate to your interested region by zoom and drag. Click on the region will generat a gray vertical indicator, and draw 'Detailed Scatter Plot' of this region below.
Detailed Scatter Plot
This panel shows the detailed plot of the variants and the genes within a certain region. Currently, only variants with -Log10(P-value) greater than 5 were displayed. The dots were colored by LD r2 value with the 'ref-variant' (If not specified, the variant with the most significant P-value was set as the default ref-variant.). The gene information tooltip provides interfaces for connecting with the gene detailed information, genome browser and eQTL signals. Please notice that the min and max plot regions of the scatter plot are 20 Kb and 1 Mb, respectively. If your input region was smaller than 20 Kb (or larger than 1Mb), the flanking 20 Kb (or 1 Mb) of the middle position was plotted.
* Trait:
FibLen_17-18
FibLen_AY-SJZ_17_r1
FibLen_YC-CS_17_r1
FibLen_XJ1-XJ2_17_r1
FibLen_XJ4-XJ3_17_r1
Region:
D11:24500000-25000000
Search
Multi Traits
Navigational Manhattan Plot (Trait: FibLen_17-18,FibLen_AY-SJZ_17_r1,FibLen_YC-CS_17_r1,FibLen_XJ1-XJ2_17_r1,FibLen_XJ4-XJ3_17_r1)
-log10 p-value
FL2
FL3
FL4
FL5
1/N: 7.13
BIN(Mb)
S01:0-0.5
A03:9-9.5
A05:108-108.5
A08:70-70.5
A11:39.5-40
A13:108.5-108.606954
D05:47-47.5
D10:30-30.5
Detailed Scatter Plot
Save PNG
Save SVG
FL2: D11 (24.51-24.78Mb)
-log10 p-value
Chromosome D11 (Mb)
FL3: A09 (61.84-62.12Mb)
Chromosome A09 (Mb)
FL4: *GhNAC49*
Gh_A10G233100
Chromosome A10 (Mb)
FL5: A07 (88.39-88.56Mb)
Chromosome A07 (Mb)
Gh_A07G212100
Gh_A07G212200
Gh_A07G212300
Gh_A07G212400
Gh_A07G212600
Gh_A07G212700
Gh_A07G213000
Gh_A07G213100

Figure S3

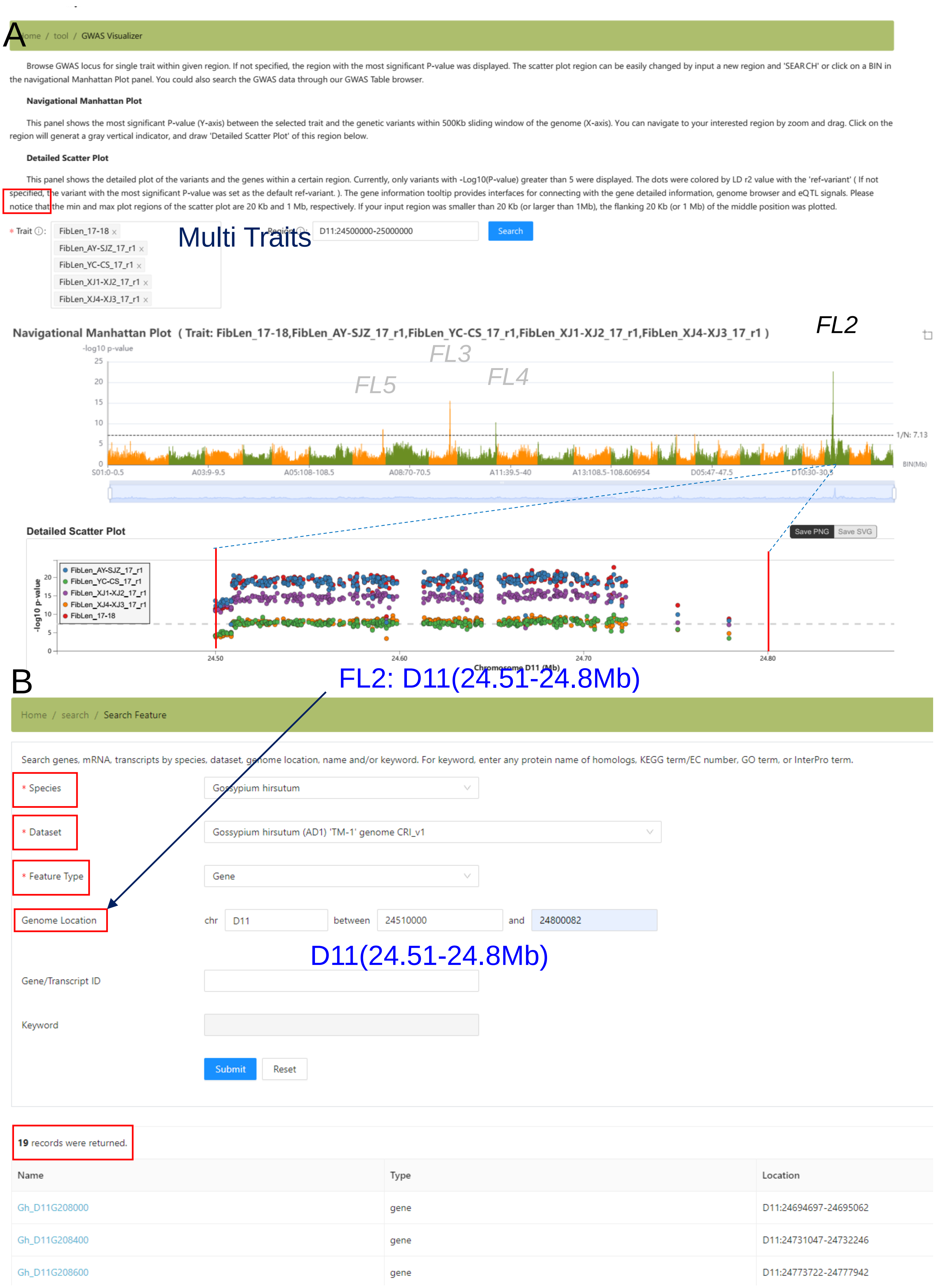
A
Home / tool / GWAS Visualizer
Browse GWAS locus for single trait within given region. If not specified, the region with the most significant P-value was displayed. The scatter plot region can be easily changed by input a new region and 'SEARCH' or click on a BIN in the navigational Manhattan Plot panel. You could also search the GWAS data through our GWAS Table browser.
Navigational Manhattan Plot
This panel shows the most significant P-value (Y-axis) between the selected trait and the genetic variants within 500Kb sliding window of the genome (X-axis). You can navigate to your interested region by zoom and drag. Click on the region will generat a gray vertical indicator, and draw 'Detailed Scatter Plot' of this region below.
Detailed Scatter Plot
This panel shows the detailed plot of the variants and the genes within a certain region. Currently, only variants with -Log10(P-value) greater than 5 were displayed. The dots were colored by LD r2 value with the 'ref-variant' (If not specified, the variant with the most significant P-value was set as the default ref-variant.). The gene information tooltip provides interfaces for connecting with the gene detailed information, genome browser and eQTL signals. Please notice that the min and max plot regions of the scatter plot are 20 Kb and 1 Mb, respectively. If your input region was smaller than 20 Kb (or larger than 1Mb), the flanking 20 Kb (or 1 Mb) of the middle position was plotted.
* Trait:
FibLen_17-18
FibLen_AY-SJZ_17_r1
FibLen_YC-CS_17_r1
FibLen_XJ1-XJ2_17_r1
FibLen_XJ4-XJ3_17_r1
Multi Traits
Region:
D11:24500000-25000000
Search
Navigational Manhattan Plot (Trait: FibLen_17-18,FibLen_AY-SJZ_17_r1,FibLen_YC-CS_17_r1,FibLen_XJ1-XJ2_17_r1,FibLen_XJ4-XJ3_17_r1)
FL2
FL3
FL4
FL5
-log10 p-value
25
20
15
10
5
0
1/N: 7.13
BIN(Mb)
S01:0-0.5
A03:9-9.5
A05:108-108.5
A08:70-70.5
A11:39.5-40
A13:108.5-108.606954
D05:47-47.5
D10:30-30.5
Detailed Scatter Plot
Save PNG
Save SVG
FibLen_AY-SJZ_17_r1
FibLen_YC-CS_17_r1
FibLen_XJ1-XJ2_17_r1
FibLen_XJ4-XJ3_17_r1
FibLen_17-18
-log10 p-value
20
15
10
5
0
24.50
24.60
24.70
24.80
Chromosome D11 (Mb)
FL2: D11(24.51-24.8Mb)
B
Home / search / Search Feature
Search genes, mRNA, transcripts by species, dataset, genome location, name and/or keyword. For keyword, enter any protein name of homologs, KEGG term/EC number, GO term, or InterPro term.
* Species
Gossypium hirsutum
* Dataset
Gossypium hirsutum (AD1) 'TM-1' genome CRI_v1
* Feature Type
Gene
Genome Location
chr D11 between 24510000 and 24800082
D11(24.51-24.8Mb)
Gene/Transcript ID
Keyword
Submit
Reset
19 records were returned.
Name | Type | Location
Gh_D11G208000 | gene | D11:24694697-24695062
Gh_D11G208400 | gene | D11:24731047-24732246
Gh_D11G208600 | gene | D11:24773722-24777942

Figure S4

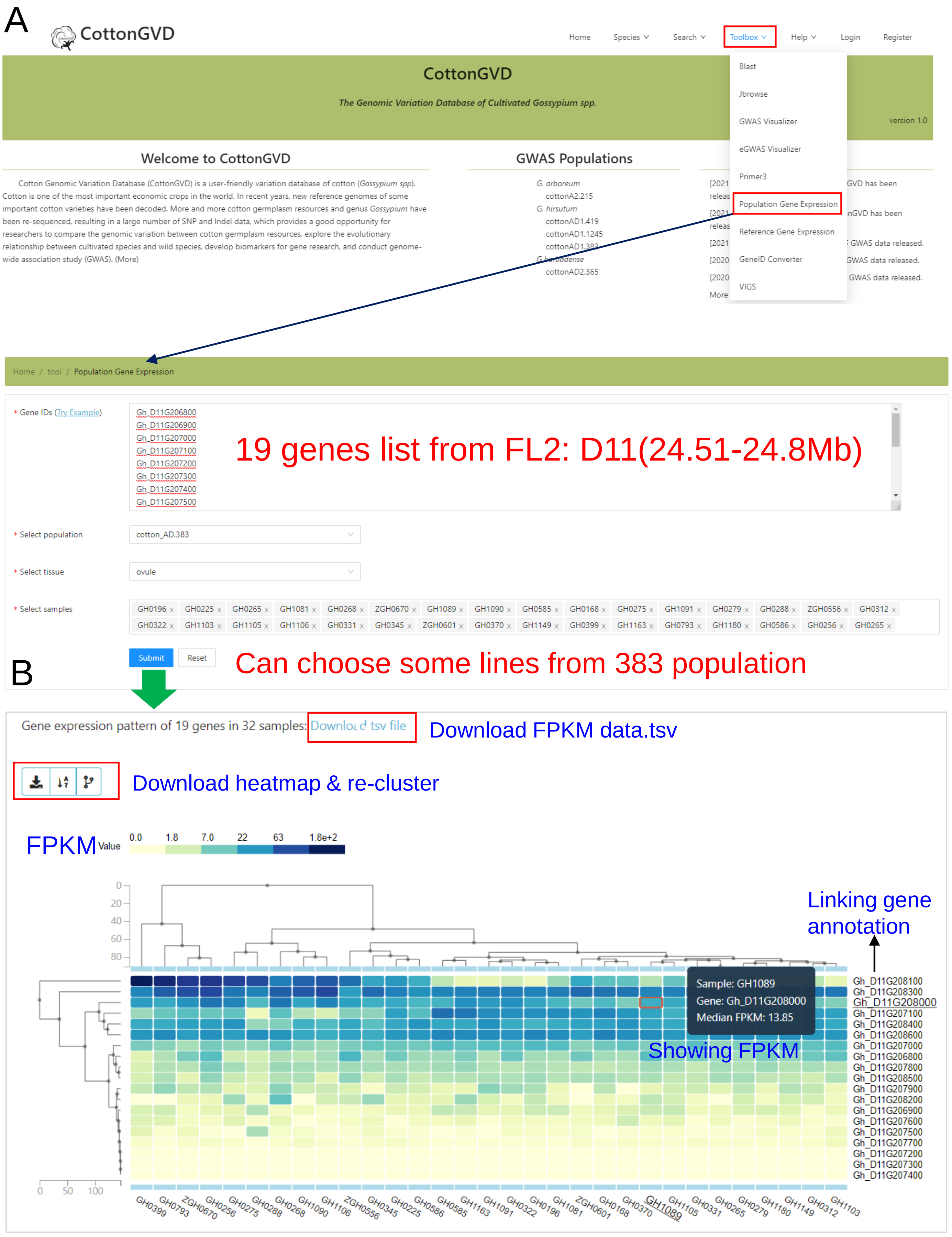

A
CottonGVD
Home
Species
Search
Toolbox
Help
Login
Register
CottonGVD
The Genomic Variation Database of Cultivated Gossypium spp.
version 1.0
Blast
Jbrowse
GWAS Visualizer
eGWAS Visualizer
Primer3
Population Gene Expression
Reference Gene Expression
GeneID Converter
VIGS
Welcome to CottonGVD
Cotton Genomic Variation Database (CottonGVD) is a user-friendly variation database of cotton (Gossypium spp). Cotton is one of the most important economic crops in the world. In recent years, new reference genomes of some important cotton varieties have been decoded. More and more cotton germplasm resources and genus Gossypium have been re-sequenced, resulting in a large number of SNP and Indel data, which provides a good opportunity for researchers to compare the genomic variation between cotton germplasm resources, explore the evolutionary relationship between cultivated species and wild species, develop biomarkers for gene research, and conduct genome-wide association study (GWAS). (More)
GWAS Populations
G. arboreum
cottonA2.215
G. hirsutum
cottonAD1.419
cottonAD1.1245
cottonAD1.383
G. barbadense
cottonAD2.365
Home / tool / Population Gene Expression
Gene IDs (Try Example)
Gh_D11G206800
Gh_D11G206900
Gh_D11G207000
Gh_D11G207100
Gh_D11G207200
Gh_D11G207300
Gh_D11G207400
Gh_D11G207500
19 genes list from FL2: D11(24.51-24.8Mb)
Select population
cotton_AD.383
Select tissue
ovule
Select samples
GH0196 GH0225 GH0265 GH1081 GH0268 ZGH0670 GH1089 GH1090 GH0585 GH0168 GH0275 GH1091 GH0279 GH0288 ZGH0556 GH0312
GH0322 GH1103 GH1105 GH1106 GH0331 GH0345 ZGH0601 GH0370 GH1149 GH0399 GH1163 GH0793 GH1180 GH0586 GH0256 GH0265
Submit
Reset
Can choose some lines from 383 population
B
Gene expression pattern of 19 genes in 32 samples: Download tsv file
Download FPKM data.tsv
Download heatmap & re-cluster
FPKM Value
0.0 1.8 7.0 22 63 1.8e+2
Linking gene annotation
Sample: GH1089
Gene: Gh_D11G208000
Median FPKM: 13.85
Showing FPKM
Gh_D11G208100
Gh_D11G208300
Gh_D11G208000
Gh_D11G207100
Gh_D11G208400
Gh_D11G208600
Gh_D11G207000
Gh_D11G206800
Gh_D11G207800
Gh_D11G208500
Gh_D11G207900
Gh_D11G208200
Gh_D11G206900
Gh_D11G207600
Gh_D11G207500
Gh_D11G207700
Gh_D11G207200
Gh_D11G207300
Gh_D11G207400
GH0399 GH0793 ZGH0670 GH0256 GH0275 GH0288 GH0268 GH1090 GH1106 ZGH0556 GH0345 GH0225 GH0586 GH0585 GH1163 GH1091 GH0322 GH0196 GH1081 ZGH0601 GH0168 GH0370 GH1089 GH1105 GH0331 GH0265 GH0279 GH1180 GH1149 GH0312 GH1103

Figure S5

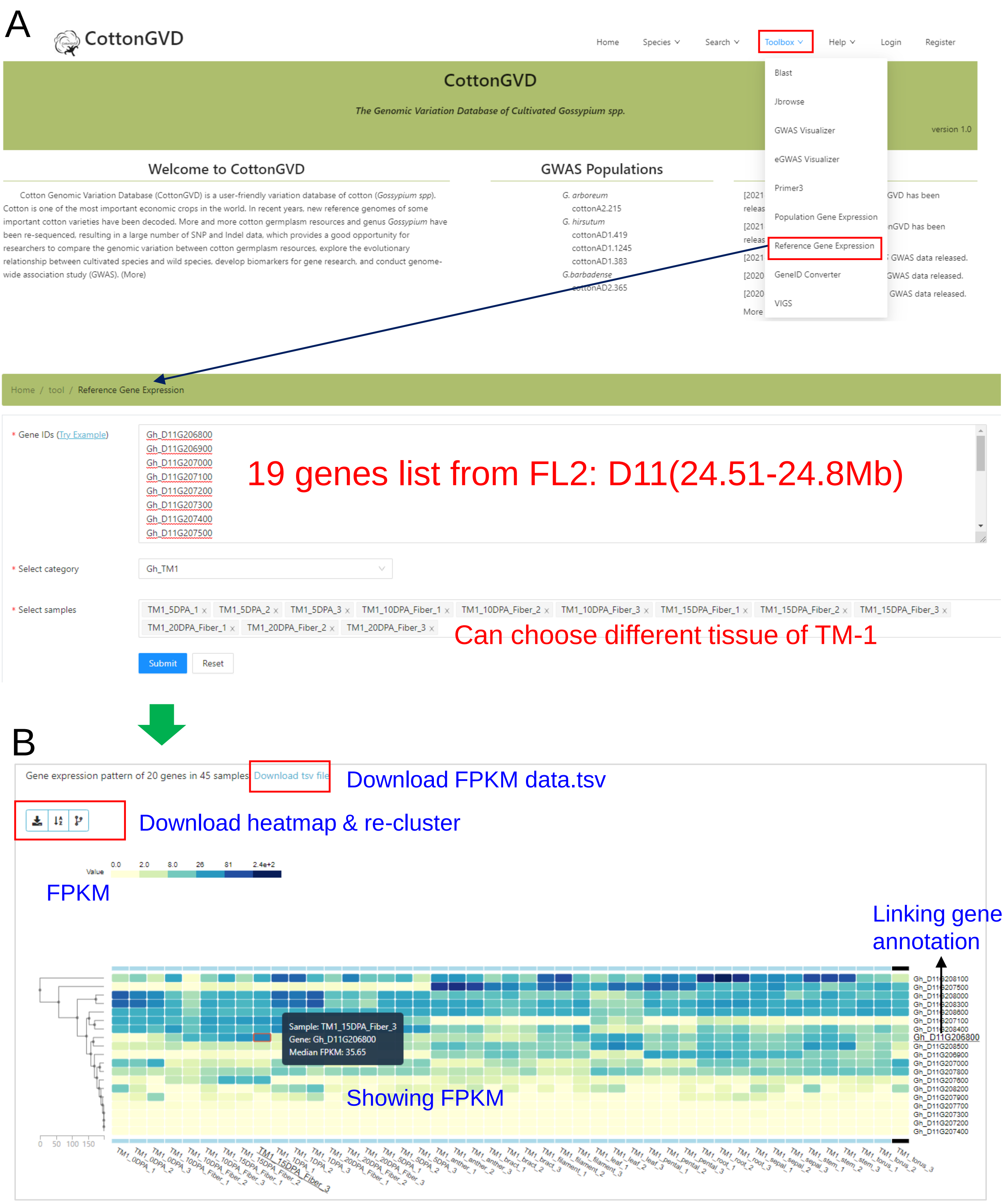
A
CottonGVD
Home
Species
Search
Toolbox
Help
Login
Register
The Genomic Variation Database of Cultivated Gossypium spp.
version 1.0
Blast
Jbrowse
GWAS Visualizer
eGWAS Visualizer
Primer3
Population Gene Expression
Reference Gene Expression
GeneID Converter
VIGS
Welcome to CottonGVD
Cotton Genomic Variation Database (CottonGVD) is a user-friendly variation database of cotton (Gossypium spp). Cotton is one of the most important economic crops in the world. In recent years, new reference genomes of some important cotton varieties have been decoded. More and more cotton germplasm resources and genus Gossypium have been re-sequenced, resulting in a large number of SNP and Indel data, which provides a good opportunity for researchers to compare the genomic variation between cotton germplasm resources, explore the evolutionary relationship between cultivated species and wild species, develop biomarkers for gene research, and conduct genome-wide association study (GWAS). (More)
GWAS Populations
G. arboreum
cottonA2.215
G. hirsutum
cottonAD1.419
cottonAD1.1245
cottonAD1.383
G.barbadense
cottonAD2.365
Home / tool / Reference Gene Expression
* Gene IDs (Try Example)
Gh_D11G206800
Gh_D11G206900
Gh_D11G207000
Gh_D11G207100
Gh_D11G207200
Gh_D11G207300
Gh_D11G207400
Gh_D11G207500
19 genes list from FL2: D11(24.51-24.8Mb)
* Select category
Gh_TM1
* Select samples
TM1_5DPA_1 TM1_5DPA_2 TM1_5DPA_3 TM1_10DPA_Fiber_1 TM1_10DPA_Fiber_2 TM1_10DPA_Fiber_3 TM1_15DPA_Fiber_1 TM1_15DPA_Fiber_2 TM1_15DPA_Fiber_3
TM1_20DPA_Fiber_1 TM1_20DPA_Fiber_2 TM1_20DPA_Fiber_3
Can choose different tissue of TM-1
Submit
Reset
B
Gene expression pattern of 20 genes in 45 samples
Download tsv file
Download FPKM data.tsv
Download heatmap & re-cluster
Value
0.0 2.0 8.0 26 81 2.4e+2
FPKM
Linking gene annotation
Sample: TM1_15DPA_Fiber_3
Gene: Gh_D11G206800
Median FPKM: 35.65
Showing FPKM
Gh_D11G208100
Gh_D11G207500
Gh_D11G208000
Gh_D11G208300
Gh_D11G208600
Gh_D11G207100
Gh_D11G208400
Gh_D11G206800
Gh_D11G208500
Gh_D11G206900
Gh_D11G207000
Gh_D11G207800
Gh_D11G207600
Gh_D11G208200
Gh_D11G207900
Gh_D11G207700
Gh_D11G207300
Gh_D11G207200
Gh_D11G207400
0 50 100 150

Figure S6

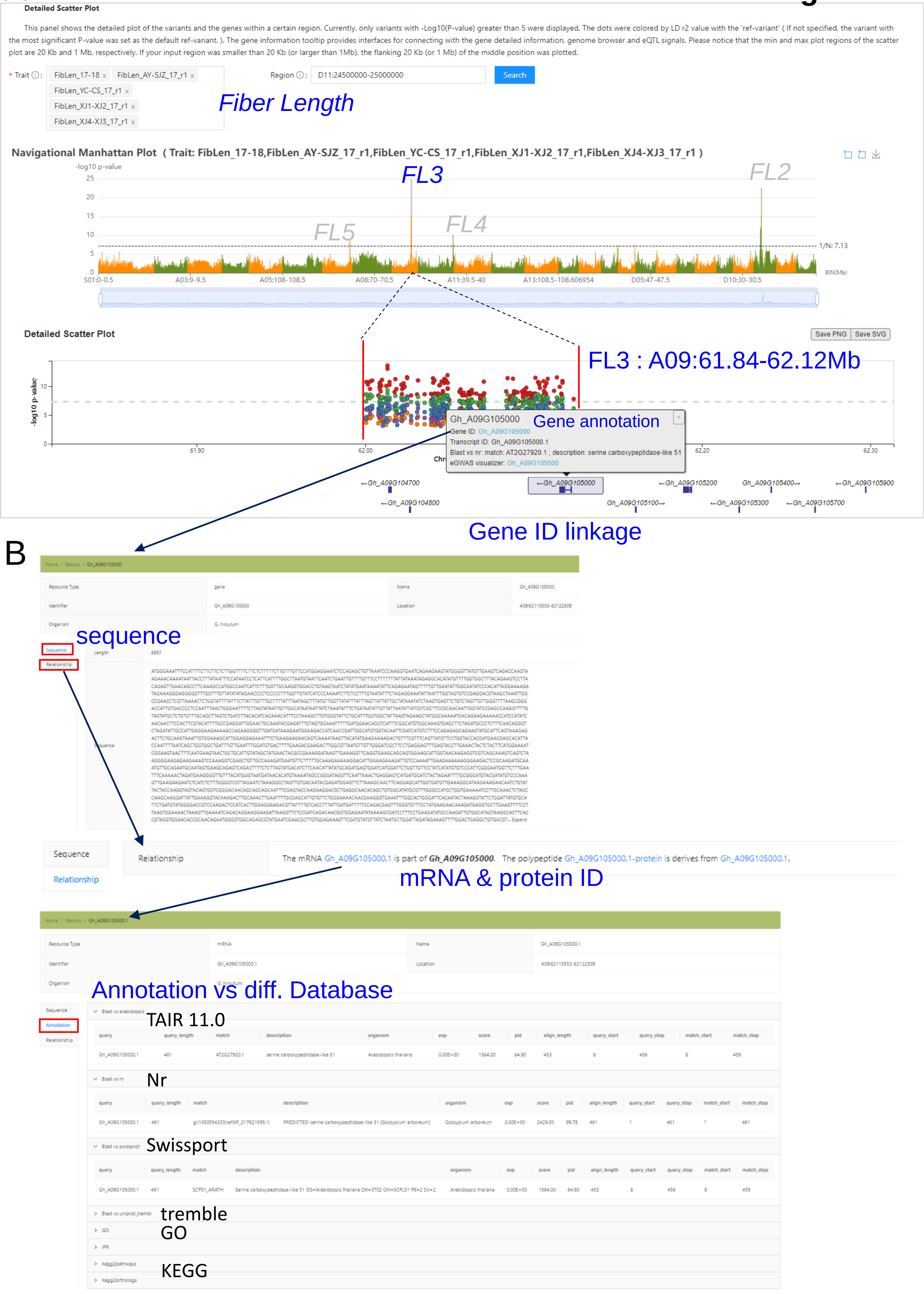
A
Detailed Scatter Plot
This panel shows the detailed plot of the variants and the genes within a certain region. Currently, only variants with -Log10(P-value) greater than 5 were displayed. The dots were colored by LD r2 value with the 'ref-variant' (If not specified, the variant with the most significant P-value was set as the default ref-variant.). The gene information tooltip provides interfaces for connecting with the gene detailed information, genome browser and eQTL signals. Please notice that the min and max plot regions of the scatter plot are 20 Kb and 1 Mb, respectively. If your input region was smaller than 20 Kb (or larger than 1Mb), the flanking 20 Kb (or 1 Mb) of the middle position was plotted.
* Trait
FibLen_17-18
FibLen_AY-SJZ_17_r1
FibLen_YC-CS_17_r1
FibLen_XJ1-XJ2_17_r1
FibLen_XJ4-XJ3_17_r1
Region
D11:24500000-25000000
Search
Fiber Length
Navigational Manhattan Plot (Trait: FibLen_17-18,FibLen_AY-SJZ_17_r1,FibLen_YC-CS_17_r1,FibLen_XJ1-XJ2_17_r1,FibLen_XJ4-XJ3_17_r1)
-log10 p-value
FL3
FL2
FL4
FL5
1/N: 7.13
BIN(Mb)
S01:0-0.5
A03:9-9.5
A05:108-108.5
A08:70-70.5
A11:39.5-40
A13:108.5-108.606954
D05:47-47.5
D10:30-30.5
Detailed Scatter Plot
Save PNG
Save SVG
FL3 : A09:61.84-62.12Mb
-log10 p-value
61.90
62.00
62.20
62.30
Gh_A09G105000
Gene annotation
Gene ID: Gh_A09G105000
Transcript ID: Gh_A09G105000.1
Blast vs nr: match: AT2G27920.1 ; description: serine carboxypeptidase-like 51
eGWAS visualizer: Gh_A09G105000
←Gh_A09G104700
←Gh_A09G104800
←Gh_A09G105000
←Gh_A09G105200
Gh_A09G105400→
←Gh_A09G105900
Gh_A09G105100→
←Gh_A09G105300
←Gh_A09G105700
Gene ID linkage
B
sequence
Sequence
Relationship
The mRNA Gh_A09G105000.1 is part of Gh_A09G105000. The polypeptide Gh_A09G105000.1-protein is derives from Gh_A09G105000.1.
mRNA & protein ID
Annotation vs diff. Database
TAIR 11.0
Nr
Swissport
tremble
GO
KEGG

Figure S6 (continue)

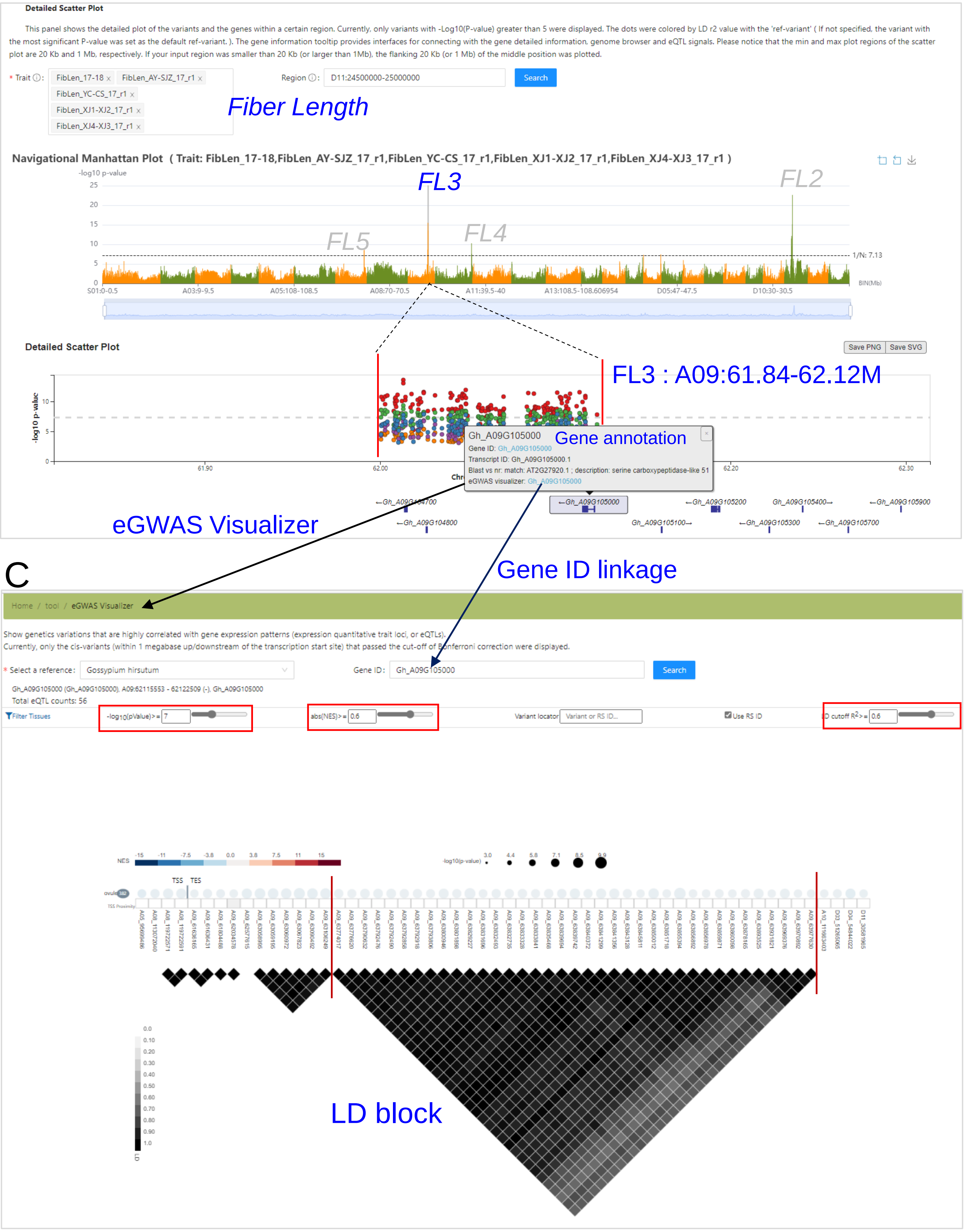

Supplement: Supplementary Figure 1 — Two ways to enter the genome-wide association study (GWAS) Visualizer tool interface are shown. [file Data_Sheet_1.PDF]
